# Project-based maturity assessment model for smart transformation in Taiwanese enterprises

**DOI:** 10.1371/journal.pone.0254522

**Published:** 2021-07-16

**Authors:** Tzu-Chieh Lin, Kung Jeng Wang

**Affiliations:** 1 Graduate Institute of Management, National Taiwan University of Science and Technology, Taipei, Taiwan; 2 Department of Industrial Management, National Taiwan University of Science and Technology, Taipei, Taiwan; University of Salento, ITALY

## Abstract

As smart technology proliferates, enterprises must engage not only in the transformation of intelligence but contend with pressure do so as soon as possible. Smart transformation is critical for manufacturing enterprises in the development of smart manufacturing. This study addressed the gap between maturity models and project management by designing an effective assessment framework for smart transformation. It adopts the Smart Industry Readiness Index, created by the Singapore Economic Development Board, as a maturity assessment model to analyze enterprises’ smart transformation and formulate project management strategies. Enterprises can use this model to examine the maturity level of their transformation and assess scope for improvement in their project strategies and implementation barriers. This study focuses on Taiwanese enterprises using data collected from 165 valid questionnaires and subjected to a cluster analysis. Enterprises were divided into three categories. The results reveal that, first, most enterprises’ smart transformation is at an immature or medium-maturity level, and is therefore amenable to further improvement. Second, inconsistent with research findings, many enterprises invest in transformation projects but fail to advance these projects to maturity. Third, most enterprises’ project management plans fail to meet actual transformation needs. Using the thematically oriented maturity model proposed in this study, Taiwanese enterprises can effectively evaluate the maturity of their transformation projects. In conclusion, the study highlights that Taiwanese enterprises must identify more effective external resources to strengthen their competitiveness.

## 1. Introduction

Since Industry 4.0, the development trend for emerging industries and disruptive technology has extended to industrial reforms through potential markets. Automation and smart manufacturing are key contributing factors in the improvement of market competitiveness. While traditional manufacturing mainly aims to develop large-scale production capacity, smart manufacturing focuses on the development of production technologies for advanced application fields. There is a growing demand for cross-domain integration and technical service customization as per consumer requirements. The strategies for smart manufacturing significantly differ from those applied in traditional manufacturing. Automation and smart application effectively reduce labor demand and considerably enhance production efficiency [1–5]. Such changes in the ecological system have led to gradual yet notable changes in the manufacturing industry. Manufacturing enterprises engage in smart transformation to increase their competitiveness and to optimize all practical applications, including operations, production, customer service, and delivery [6, 7]. Smart transformation introduces new ways for enterprises to present their products in the market. Smart transformation has become the key to measure the enterprise capability and competitiveness. It involves organization design [8], dynamic capability identification [9], business model establishment [10, 11], discovery of new strategies [12], and seizing market opportunities by introducing new digital technologies. It is also defined as "all aspects of business and society that fundamentally affect business and society by applying digital technology" [13]. Instead of focusing on a single product, service, or technology, it is committed to cross data and cross domain transformation. In fact, with the development of digital technology, the components of perception, thinking, connection and action ability have become the key to smart transformation.

However, enterprises planning for transformation, particularly project managers in charge of technology, budgets, or development, are faced with various uncertainties [14]. While enterprises have been pursing transformation for several decades, many have failed to transform successfully, and some have even claimed bankruptcy. Established organizations such as Nokia Corporation, The Eastman Kodak Company, General Electric Company, and Koninklijke Philips N.V. seem to be diminishing in their influence. Similar trends have been reported for Tsann Kuen and Datong enterprises in Taiwan, which were once the leading information technology firms.

Thus, transformation is not an end but a means to strengthening an enterprise’s competitiveness and is certainly not free from risks.

From the perspective of project execution, it is necessary to design a reliable and valid tool that can help project executives evaluate transformation levels and monitor project progress to reduce potential risks during the transformation process.

In 2021, the manufacturing industry is likely to experience a shortage in factory labor and reduced output and capacity owing to the Covid-19 pandemic and the trade dispute between the United States and China. To reduce dependence on labor, enterprises have been accelerating the upgradation of manufacturing automation and intelligent systems [15]. Given the inevitable growth in demand for smart manufacturing, enterprises must not only invest in automated systems [1] but also implement effective project management plans and transformation strategies to tackle unpredictable external risks. The resulting development of various digital, smart technologies has rendered the role of project management and transformation planning increasingly important.

Taiwan’s manufacturing industry was initially driven by production but gradually turned its focus to smart manufacturing to keep up with global market competition. Taiwanese manufacturing enterprises have begun investing in smart manufacturing projects to gradually introduce digital technology while maintaining original equipment manufacturing capacity and retaining electronic manufacturing services (EMS).

Deloitte [16] conducted a survey on enterprises, dividing them into three groups: new entrants, leaders, and followers. Their results reveal that smart transformation readiness is considerably low among the sub-sectors of the manufacturing industry, with semiconductor companies accounting for the highest proportion. Most smart transformation leaders have exceeded consumer requirements, and thus, they face higher pressure from their competitors than from their customers. In addition to such pressure, the industry is confronted by not only the costs of replacing old machines with smart ones but also the uncertainty of smart transformation. The transformation of business scope and operations into smart manufacturing is key in complex project planning, budget considerations, and resource management [7]. These conditions make it increasingly difficult for Taiwan’s manufacturing industry, which is predominantly composed of small- and medium-sized enterprises (SMEs), to keep pace with smart transformation. Smart manufacturing is challenging because numerous advanced technology projects must be completed in the production environment. At this transformation stage, the roles of project management involve more dynamic production environments and components, including products, people, and machines. Existing research on maturity assessment models for smart transformation projects and management is insufficient for effecting smart transformation. Because the optimization of manufacturing capabilities is integral to adapting to a fast-changing manufacturing environment, enterprises should seek an effective maturity model for implementing smart manufacturing transformation. Thus, this study uses a maturity assessment model to analyze Taiwanese enterprises in terms of resource availability. In addition, it modifies the Smart Industry Readiness Index created by Singapore’s Economic Development Board by adding the dimension of project management to form a new thematically oriented maturity model.

This study is guided by the following research questions:

RQ1: How does project management affect smart manufacturing transformation and how can be it incorporated in the maturity assessment model as an indicator of enterprise transformation?
RQ2: How can project management be incorporated in the maturity model as a self-assessment measure to improve enterprises’ smart manufacturing strategies?

We address the abovementioned research questions by examining the relationship among transformation projects, strategies, equipment, processes, and organizations and compare the maturity model in this study with those in the literature.

The rest of this article is organized as follows. Section 2 reviews the literature on smart innovation, transformation projects, and maturity models for manufacturing enterprises. Section 3 describes the research methods adopted to develop the maturity model and applies the model to 165 enterprises in Taiwan. Section 4 presents the investigation results as well as the cross-domain findings of a cluster analysis. Section 5 analyzes and discuss challenges faced in the transformation process. Section 6 offers conclusions and possible applications of the model in the manufacturing industry and discusses the limitations of this research and the next steps for enterprises to cope with changing market demands.

## 2 Literature review

### 2.1 Smart transformation in enterprises

In the era of intelligent system, various technology applications can be used to strengthen the scientific and technological capabilities of enterprises. Smart manufacturing is a technology driven method. It uses the new generation of IT applications such as cloud-computing, big data, machine learning, artificial intelligence (AI), Internet of things (IoT), etc., so that every link in the production process is highly customized and intelligent, enabling manufacturers to respond to the rapidly changing market demands faster [17–19]. Therefore, the adoption of advanced technologies such as cyberphysical systems (CPS) is critical for the implementation of smart manufacturing [17, 19, 20]. It is emphasized that the end-to-end digitization of all physical applications is realized by introducing the digital value chain system, the horizontal integration of value network and the vertical synthesis of manufacturing system [17, 21].

Smart transformation provides a key driver for manufacturing to smart manufacturing in particular [17, 20]. It promotes the smart transformation of enterprises and helps them develop new business models to improve products, organizational structure, or processes [9, 22], information and knowledge sharing [23], and seize market opportunities [11]. External knowledge impact on innovation performance and transformation [24]. They are reshaping manufacturing and driven by smart transformation [25]. It is essential to implement smart manufacturing [17, 20, 23, 26, 27].

According to the report of MarketsandMarkets, the potential market value of smart manufacturing is expected to reach US $1566.6 billion in 2024 [28]. It is expected that by 2025, the global digital transformation market will reach US $1009.8 billion [21], of which North America is the main digital transformation market. The EU also proposed a "digital Europe plan" to accelerate the development of digital transformation [29]. Many other countries (such as the Japan, Korea, China, and United States) have also proposed various smart transformation initiatives to take advantage of this market opportunity, especially in the field of smart manufacturing [26].

Traditional manufacturing industry used to provide single service or product supply and established competitive OEM/ODM industrial base with low cost and flexible advantages. However, with the transformation of global industrial structure, the traditional business model has gradually changed to customer and service oriented. To keep up with this trend, enterprises must think digitally and emphasize the use of digital technology to expand product vision and build up new business models [8, 30]. When manufacturing enterprises implement transformation projects, their battle is no longer limited to redesign products, improve efficiency or improve profitability, but focus on promoting high added value, and seize new opportunities and lead the industry with existing expertise [15]. In addition, the smart transformation must also consider internal optimization operations and external response to competitors, which will improve awareness and dynamic response capabilities in the competitive environment [31]. Therefore, it is very important for manufacturing enterprises to understand the effective implementation of "transformation project".

Traditionally, the implementation of smart transformation of enterprises can be divided into strategies, organizations, processes, and other aspects. After accumulating certain project resources and experience, enterprises begin to transform hand in hand. The transformation process can be divided into short-term inventory management, medium-term technology incubation, 5–10 year prediction according to the time, and it can be divided into different blueprints according to the enterprise blueprint. Prahalad and Oosterveld [32] put forward five factors that influence transformation. Firstly, enterprise must focus on new opportunities, not just reducing costs, improving profitability, or redesigning products. Secondly, the top managers of the enterprise must lead the transformation project. Thirdly, the transformation project must have a clear vision. Fourthly, transformation projects must aim at developing new organizational capacities and skills to maintain competitiveness. Finally, the transformation project should be incorporated into the new management strategy and process to achieve changes in performance, remuneration, occupation, and product development. Therefore, the realization of smart transformation requires not only the development of new value in the market, but also the improvement of enterprise profits. To achieve this goal, enterprises must have innovative capabilities, create new business models, implement transformation projects, and finally realize smart manufacturing.

### 2.2 Smart manufacturing and project management

Responsibilities in project management include planning, managing, organising, and scheduling project activities. Project management is composed of knowledge, skills, and tools applications to meet the project requirements [33]. Projects differ in terms of the five ‘value drivers’, namely 1. market payoff, 2. project budget, 3. product performance, 4. market requirement, and 5. project schedule [34].

With the rapid spread of digital technologies in Industry 4.0, accelerating smart transformation in industries is the most critical challenge. In addition, smart manufacturing projects have implemented many technologies, such as automation, industrial Internet of Things (IIoT), sensing technology, big data analysis, and cloud-computing application [17], and thus, relevant solutions must be introduced for different production lines. In the process of transforming from traditional manufacturing to smart manufacturing, business scope and operations are transformed into smart manufacturing, and enterprises are faced with complex project planning, budget consideration and resource management process [7].

From the perspective of organizational operations, transformation impacts an enterprise’s business model and value. Thus, enterprises must be supported by senior-level executives to create a broad vision for strategies, organization, operations, and products [35]. Moreover, the senior management team must fully understand the resources and capabilities of the enterprise before they are qualified and able to be responsible for the implementation of the optimized transformation project of the enterprise. Next, project managers or project leaders adopt assessment tools to measure the strengths and weaknesses of the enterprise and assess the level of maturity to improve the capabilities required for project implementation. Wang et al. [36] also reported that the level of confidence of project managers would affect the implementation of the project. Liu et al. [37] discovered that Integrated Project Delivery (IPD) affect the value of project, and project management can cope with different project attributes [38]. In terms of manufacturing, its project management is closely related to technology management [39].

The steps to be taken when the transition project is in progress are as follows. First, we should form a vision of smart manufacturing projects. Next, a project assessment should be conducted to identify possible problems and then a cross functional project team should be established [40–42]. In the category of smart transformation, the best ideas and strategies for smart manufacturing are introduced by the project team through a close network [19, 26, 41, 43]. Its relevant team consists of members from the production and IT department and the technology development department [18, 41, 42, 44, 45]. It also considers that it involves a variety of professional technologies and uses reliable evaluation tools to realize the smart transformation of manufacturing enterprises.

Research has investigated smart manufacturing project management and application [46–49]. Win and Kham [46] developed a framework for project management, manager roles in Industry 4.0, and algorithms to trace project data through a CPS [47]. Dreyer et al. [48] advanced a project portfolio selection decision model to evaluate investment in infrastructure projects and production entity projects in Industry 4.0. Cakmakci [49] emphasized project management as a critical tool in Industry 4.0. Although these studies have offered project management methods to assist enterprises in transitioning to smart manufacturing, these methods have involved complex processes, lacked pack execution assessment tools, required extensive capabilities, and excluded other relevant transformation projects for introducing smart manufacturing. These qualities present a research gap in project management and smart manufacturing transformation.

When introducing projects, enterprises should also consider the impact of smart manufacturing on the production process, technological developments, and overall infrastructure. During our literature review, we concluded that smart manufacturing project management employs varying management methods and technical skills to enhance the efficiency of a project. Smart manufacturing project management differs from traditional project management because of a greater need for technological evaluation. As a result, project management in smart manufacturing is critical for project success.

### 2.3 Maturity assessment model

Smart manufacturing transformation projects are developed using various digital technologies, and thus, they must be evaluated at multiple levels. An evaluation system helps transform an abstract concept into practical application tools. The maturity model will enable enterprises to determine their capabilities and systematically realize their vision of smart manufacturing. Santos and Martinho [50] report that maturity model is a tool to measure the maturity level of enterprises to achieve their ultimate goals. It can also be used to evaluate the initial development stage of the enterprise and to develop the future development strategy [51]. The paper thinks that project managers should adopt maturity model to evaluate the different levels of project management, equipment efficiency, process improvement and organization management, so as to overcome their weaknesses and promote the transformation of smart manufacturing. Schumacher et al. [52] pointed out that maturity model is the appropriate tool to assess the level of resources of an organization and helps organizations take appropriate action. Backlund et al. [53] asserted that maturity models are essential in the evaluation of organizational standards. Maturity model can evaluate the capability of enterprises and establish the path to implement smart manufacturing strategy [35]. Zvetko et al. [54] proposed an Industry 4.0 assessment model for regional development. The above research shows that various maturity models or assessments (see Table 1) have certain validity on the maturity level of smart manufacturing [6, 7, 52, 54–64].

**Table 1:** Overview of maturity models.

Model Name	Structure	Applicable Industry	Business Scope	Assessment of Project	Reference
The Connected Enterprise Maturity Model	5 levels of models with 4 dimensions	IT- capability of enterprise	Not specific to SME	Not Considered	Rockwell Automation [7]
(Industry 4.0) Maturity Model	5 levels of models with 9 dimensions	Manufacturing Industry	Not specific to SME	Not Considered	Schumacher et al. [52]
IMPULS (VDMA) (Industry 4.0 Readiness)	5 levels of readiness and 6 dimensions	Manufacturing & engineering	Not specific to SME	Not Considered	Lichtblau, et al. [59]
Three stage maturity model	3 maturity of model	All industries	Considered	Not Considered	Ganzarain and Errasti [57]
Industry 4.0 (Digital Operation Self-assessment)	4 level of maturity, 6 dimensions	Specific industry	Not specific to SME	Not Considered	PWC [6]
M2DDM	6 level of maturity	Manufacturing Industry	Not specific to SME	Not Considered	Weber et al. [64]
DREAMY -Digital Readiness Assessment Maturity	4 dimensions	Manufacturing Industry	Not specific to SME	Not Considered	Carolis et al. [56]
Industry 4.0-MM	7 MM (maturity model) 5 characteristics	Manufacturing Industry	Not specific to SME	Not Considered	Gökalp et al. [58]
MPI	6 stages of digital maturity	All industries	Considered,	Not Considered	MPIG [61]
Singapore Index	3 circles, 3 blocks and 8 pillars	All industries	Considered	Not Considered	Singapore EDB [62]
Industry 4.0 Maturity Model—MM	7 dimensions and 38 maturity items	auto-component manufacturing	Not specific to SME	Not Considered	Wagire et al. [63]

Maturity model is suitable for the transformation of smart manufacturing. Canetta et al. [65] reported that most studies have used standard models for digital maturity assessments, but these models do not illustrate the practical process of transformation. Basl [55] pointed out that using the model to assess maturity can be done at both the macro (social or national) and micro (enterprise) levels. PwC [6] provides an online tool to assess the level of maturity of the enterprise to determine the next step of improvement actions. Most studies describe the degree of readiness to build maturity models or evaluation methods to assess the degree of readiness for enterprise transformation. From Table 1, the majority of the literature describes how to construct an assessment method to measure the smart readiness of manufacturing enterprises. Manufacturing enterprises perform the steps to review their level of smart readiness and explore their opportunities for smart transformation. However, the existing models are designed for applications in large enterprises and take SMEs only partially into consideration when examining organisational dimensions. These models do not explain or design a suitable strategy path for project guidance or transformation project in the smart transformation procedure after assessing maturity. Hence, the development of a project-based framework is necessary to fill the research gap.

Though these methods, enterprises can reexamine their own weak links and improve the opportunities for transformation.

## 3. Quantitative study

**Fig 1:**
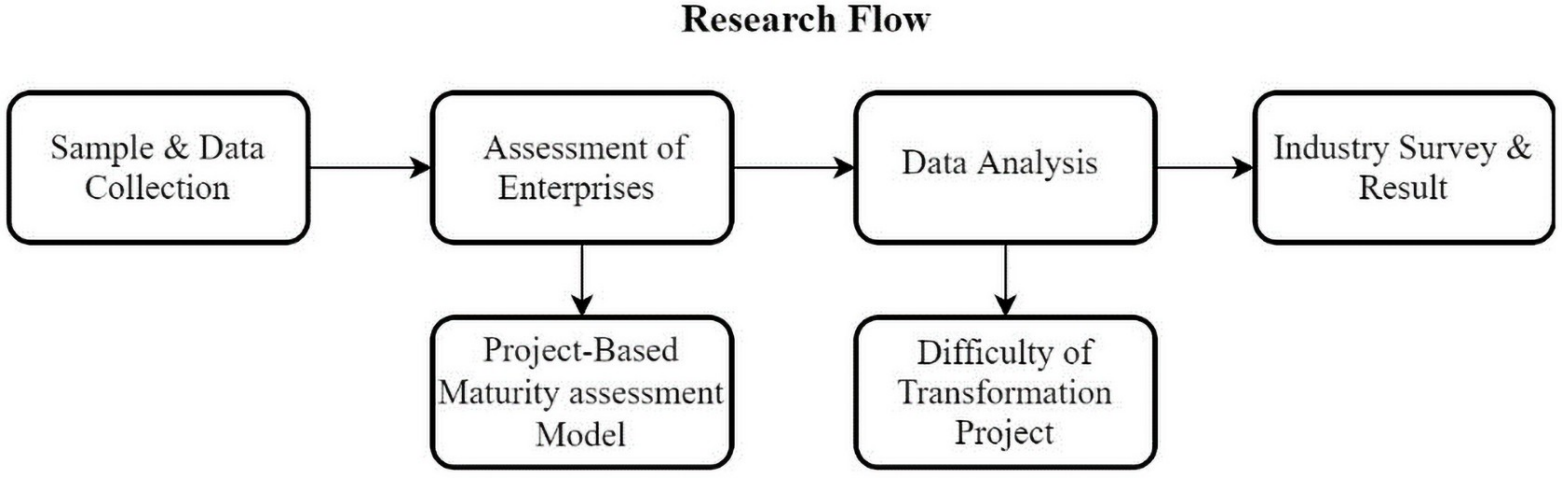
Research flow.

This study uses the project-based maturity model to make a statistical analysis of the maturity of Taiwan enterprises. Then, it investigates the difficulties encountered in the transformation project, analyzes the implementation obstacles, and determines the key factors of the project according to the opinions of senior managers. Fig 1 shows the research flow.

### 3.1 Singapore index

**Fig 2:**
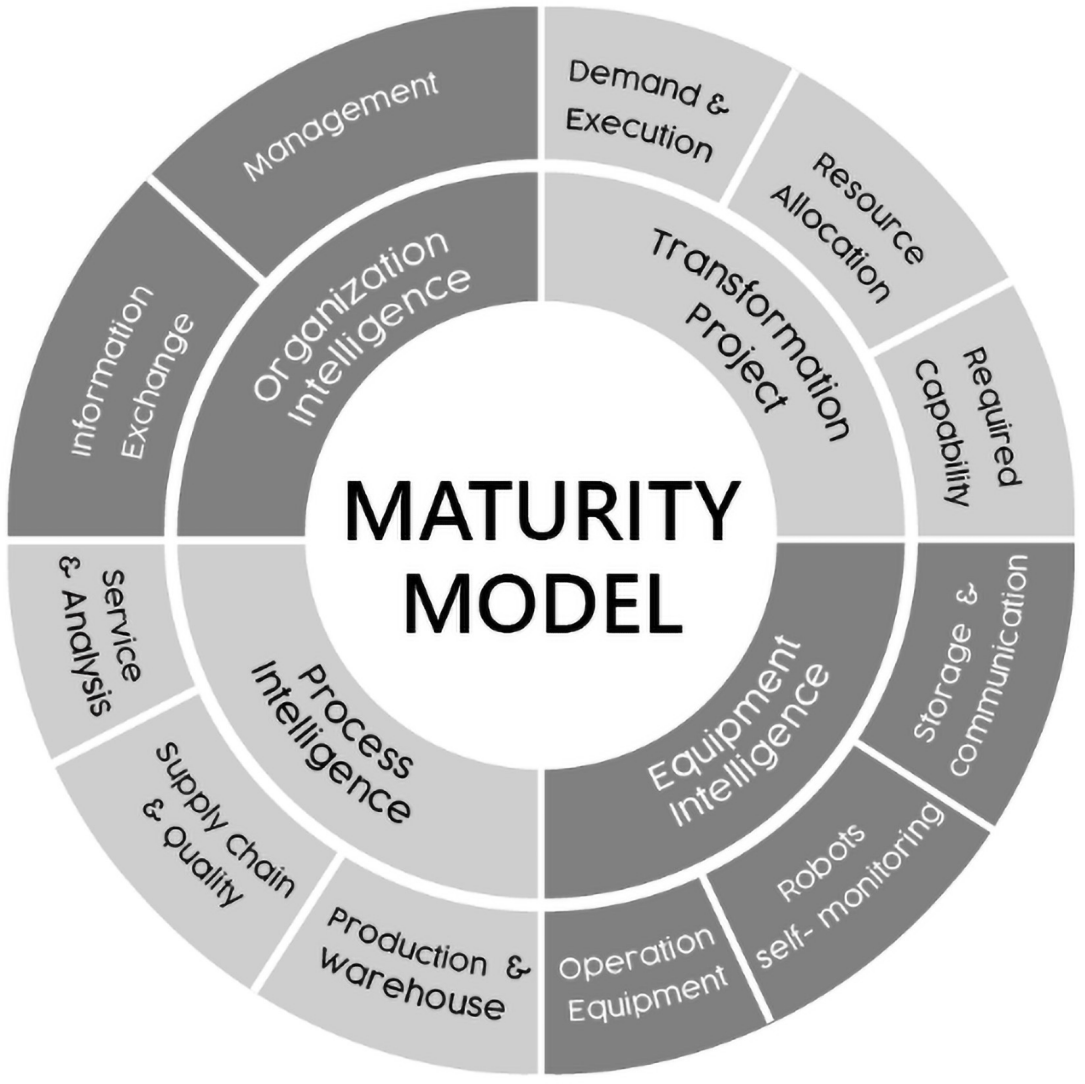
Proposed project-based maturity model (modified from Lin et al. [20]; Singapore EDB [62]).

Singapore index [62] is a convenient method to evaluate the maturity of enterprises. The advantages of the maturity assessment model are that it evaluates various dimensions and is easy to be used for the actual assessment of government sponsored Multinational Corporation (MNC) and Small and Medium Enterprises (SME) [20]. Another characteristic of this maturity evaluation model is that it is suitable for the evaluation of Taiwan enterprises, promotes continuous improvement and has a wide range of industrial applications [20]. But Lin et al. [20] also suggests tools to identify next steps in the transformation project to enhance the ability of enterprises to guide them to achieve smart manufacturing transformation. This paper proposes a new topic-oriented maturity assessment model based on the framework of Singapore maturity model, which combines the functional areas of project management and transformation projects, including requirements and implementation, resource allocation and required capabilities. We divided our model into four core areas, of which three originate from the Singapore maturity model: (1) equipment intelligence; (2) process intelligence; (3) organizational intelligence; and (4) transformation project, the new element in our model. Therefore, we separated core content and related applications into four functional blocks (Fig 2).

Fig 2 illustrates the proposed model, including four internal loops: transformation project, equipment intelligence, process intelligence, and organizational intelligence. The architecture of the new model consists of four functional blocks and 11 key pillars that measure the different levels of application of the enterprise for practical evaluation. The new assessment model focuses on each stage of technology development and the required capabilities and helps to identify follow-up actions to improve enterprise value. In addition, similar to Singapore maturity model, the new model is validated by applying it to enterprises with different business models, including SMEs and transnational corporations [62]. Therefore, the main purpose of this study is to propose a project-based maturity assessment model to evaluate the maturity level of Taiwan enterprises and verify its application in practical application.

### 3.2 Sample and data collection

To assess enterprises’ maturity level, an online survey was conducted among senior executives, operation managers, and project managers from the IT, engineering, or manufacturing fields. Data were collected from 165 Taiwan-based enterprises, and the respondents were asked to rate questionnaire items on a five-point Likert scale. This questionnaire is anonymous. If you agree to complete this questionnaire, you acknowledge that you have understood the relevant details of this research and consent to participating. Respondent contact information (including email addresses) is collected in accordance with Taiwan’s Personal Information Law. These enterprises were classified into the technology industry category based on their technological intensity, according to the statistical classification of economic activities in the European community, (NACE Rev 2) [29]. They were categorized into high-technology, medium-high-technology, medium-low-technology, and low-technology industries (Table 2). In addition, traditional manufacturing enterprises with fewer technological features and who were in the process of introducing advanced solutions were surveyed. At the time of the survey, a majority of the enterprises’ smart manufacturing projects were either in the planning or implementation stage. These surveyed enterprises included SMEs, MNCs, and group-based units, with some having their own production lines or related manufacturing facilities in Taiwan and overseas. All constructs demonstrated appropriate reliability and validity.

**Table 2:** Classification of manufacturing industries based on NACE Rev. 2 [66].

Number	Classification	Description of Industry
1	High technology	Manufacture of basic pharmaceutical products and preparations
		Manufacture of computers, electronics, and optical products
		Manufacture of aircraft, spacecraft, and related machinery
2	Medium high technology	Manufacture of chemicals and chemical products
		Manufacture of weapons and ammunition
		Manufacture of electrical equipment
		Manufacture of machinery and equipment n.e.c.
		Manufacture of motor vehicles, trailers, and semitrailers
		Manufacture of other transport equipment, excluding ships and boats, and manufacture of aircraft, spacecraft, and related machinery
		Manufacture of medical and dental instruments and supplies
3	Medium-low technology	Manufacture of coke and refined petroleum products
		Manufacture of rubber and plastic products; other nonmetallic mineral products; basic metals; and fabricated metals products, except machinery and equipment
		Repair and installation of machinery and equipment
4	Low technology	Manufacture of food products, beverages, tobacco products, textiles, apparel, leather and related products, wood and wood products, paper and paper products, printing and reproduction of recorded media
		Manufacture of furniture, other manufacturing

According to the model classification, five definitions of maturity level and status were proposed (Table 3). The maturity model enables enterprises to re-evaluate or assess their transformation status and capabilities for further enhancement. The proposed model helps determine five levels of maturity (1–5) in the project, equipment, process, and organization aspects: level 1 (initiated) has a score range of 1–26, level 2 (performed) has a score range of 27–53, level 3 (managed) has a score range of 54–80, level 4 (optimized) has a score range of 81–107, and level 5 (implemented) has a score range of 108–130. This study administered 26 questionnaires for the survey; hence, the highest accumulated maturity score was 130 (26 × 5) (Table 3). Furthermore, levels 1 and 2 were defined as immature levels, levels 3 and 4 as mid-mature levels, and level 5 as a completely mature level. The scores were aggregated to obtain the overall value of the maturity level.

**Table 3:** Status and definition of maturity levels [6, 9, 20, 22, 56–58].

Level	Status/Score	Definition
1	Initiated (1–32) (Immature)	• Initial demand assessed and transformation plan evaluated
		• Project, equipment, process, and organization aspects in early stages
		• Project management capabilities assessed and issues identified
2	Performed (33–65) (Immature)	• Transformation plan partially developed and presented with required capabilities
		• Project, equipment, process, and organization aspects established and executed
		• Internal and external resources evaluated to enable change in plans according to project capability
3	Managed (66–98) (Med-mature)	• Transformation plan well executed
		• Project, equipment, process, and organization ongoing and standardization achieved
		• Data analytics and system integration partially achieved Standard process for capability improvement established
4	Optimized (99–131) (Med-mature)	• Optimization of transformation plan achieved
		• Project, equipment, process, and organization aspects at the integration and interoperability stage
		• Smart (digital) transformation launched
		• All resources for improvement of project capability integrated
5	Implemented (132–160) (Mature)	• Transformation plan actively completed
		• Project, equipment, process, and organization aspects achieved the digital manufacturing ecosystem and have been assessed to have reached a high maturity level
		• Smart (digital) transformation implemented
		• CSF and lead capability improvement implemented in the project

## 4. Research analysis

### 4.1 Data analyses and results

This study classified the respondents into high-technology (59%), medium-high-technology (21%), medium-low-technology (11%), and low-technology (9%) groups based on NACE version 2 [66]. In total, 45% of respondents were from large-scale enterprises and 55% were from SMEs. Of the respondents, 69% were senior executives (including CEOs, GMs, and VPs); 17% were in the IT, engineering, or operation positions; and others (14%) were in project-related positions. The respondents’ work experience certainly contributed to the quality of this study. On average, they had work experience of over 13 years in engineering, project management, production management, and senior executive operations in the surveyed companies.

**Fig 3:**
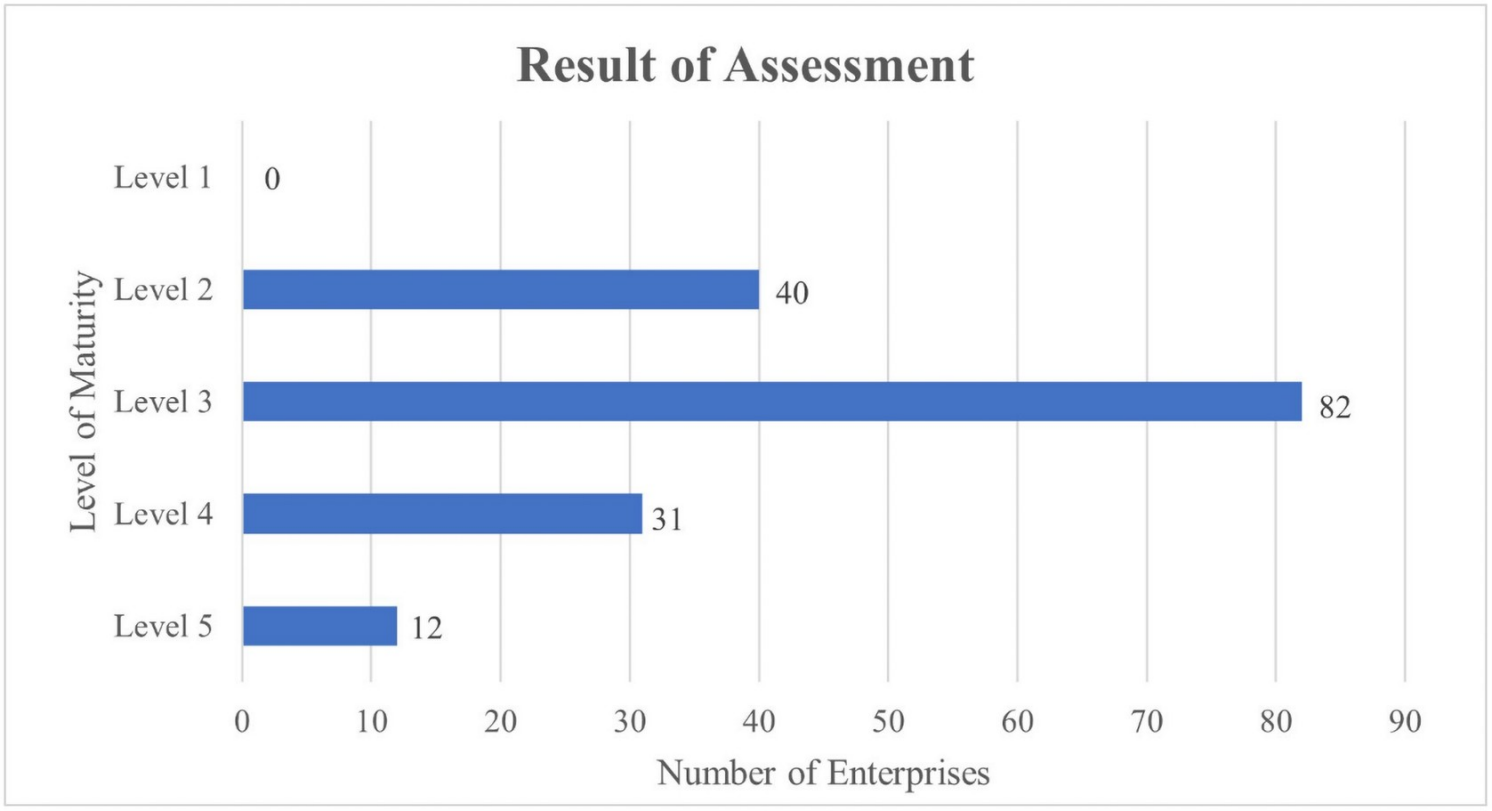
Overall maturity levels of enterprises.

Of the total number of firms surveyed, 40 were immature (0 at level 1 and 43 at level 2), 113 were mid-mature (82 at level 3 and 31 at level 4), and 12 were mature (level 5; Fig 3). This finding indicates that Taiwanese enterprises intensively invested in smart manufacturing transformation, and the number of mid-level mature and mature enterprises increased; moreover, enterprises invested in transformation plans or executed the plan from 2018 to 2021. Our study results revealed that most Taiwan-based enterprises were in the transformation plan planning and execution stages in 2021, whereas Lin et al. [20] indicated that most Taiwan-based enterprises were still in the beginning or planning stages in 2018. Thus, Taiwan’s manufacturing industry has undergone major changes in the past 3 years, especially in terms of investment in smart manufacturing transformation projects. The structure of the manufacturing industry has obviously changed, which has promoted the upgrade of the entire industrial supply chain system to a new ecosystem of smart manufacturing.

Table 4 presents the contributions of the project, equipment, process, and organization aspects to smart transformation. The descriptive statistics indicate that the dispersion of the process dimension was higher than that of the other dimensions among the groups, with a higher concentration.

**Table 4:** Descriptive statistics (mean and standard deviation).

	Mean	S.D.	N
Project	13.0424	5.11854	165
Equipment	16.0545	5.56585	165
Process	8.5152	2.95413	165
Organization	35.0909	11.00045	165

Table 5 illustrates K-means cluster analysis results for the classification of the 165 surveyed enterprises into three groups.

**Table 5:** Cluster analysis of groups: Group categories and industry analysis.

Final Clustering Center			
Grouping	1	2	3
Project	37.45	23.78	50.50
Equipment	12.93	9.75	19.38
Process	16.53	11.93	22.56
Organization	8.86	6.24	11.91
N	74	59	32
Industry Analysis	System Integration, Design-based SME, Mechanical Equipment, EMS (SME)	Traditional manufacturer (Parts, Component, Accessory, Plastic, and metal processing)	EMS, Information service provider, Automation

This study adopted a k-means clustering algorithm for further grouping analysis. A significant gap was observed between group 2 (59 enterprises) and group 3 (32 enterprises). We analyzed the enterprises in groups 2 and 3 and found that group 2 comprised traditional manufacturing enterprises supplying parts, components, accessories, plastic and metal processing and had no immediate plans to execute any transformation project owing to production or manufacturing factors. In group 3, the highest values of the domains were obtained and included industries dealing with automation, information service provider, and large-size EMS. For the grouping, the transformation plan was optimized and the relevant capabilities were integrated for interoperability stages.

A small gap was observed between group 1 (74 enterprises) and group 2 (59 enterprises). The enterprises in group 1 are in the business model of system integration, design-oriented SMEs, and mechanical equipment. The enterprises in this group provide integrated-to-use, or customized solutions. Further analysis revealed that group 1 exhibited higher values in the project, equipment, and organization domains with a higher degree of active planning and execution compared with group 2. Most enterprises in group 3 were large EMS, automation and some were solutions and service providers. In this group, the enterprises have implemented transformation projects and standardized an improved plan for enhancing capabilities. To sum up, according to our analysis, group 3 had the highest maturity, but enterprises in this group need to strengthen their capabilities in the four domains and move toward smart manufacturing.

Pearson’s correlation was used to compare relationships between variables, and it indicated moderate correlations among project, equipment, process, and organization (Table 6), which were satisfactory for all constructs.

**Table 6:** Pearson’s correlation results.

		Project	Equipment	Process	Organization
Project	Pearson’s Correlation	1	.620**	.619**	.668**
	Sig.(2-tailed)		0.000	0.000	0.000
	N	165	165	165	165
Equipment	Pearson’s Correlation	.620**	1	.595**	.563**
	Sig.(2-tailed)	0.000		0.000	0.000
	N	165	165	165	165
Process	Pearson’s Correlation	.619**	.595**	1	.645**
	Sig.(2-tailed)	0.000	0.000		0.000
	N	165	165	165	165
Organization	Pearson’s Correlation	.668**	.563**	.645**	1
	Sig.(2-tailed)	0.000	0.000	0.000	
	N	165	165	165	165

Note:

‘**’ denotes significance at the 0.01 level (two-tailed).

### 4.2 Validity and reliability

Validity and reliability tests were conducted to measure biases and distortions. Table 7 presents Cronbach’s α [67, 68] values that represent the reliability in this study; values in the project, equipment, and organization domains exceeded 0.90 (i.e., 0.922), indicating high reliability; moreover, the value for the process domain was acceptable. Furthermore, Nunnally [69] reported that a reliability coefficient of 0.70 is sufficient.

**Table 7:** Reliability and validity test. (a) Case processing summary (b) Reliability statistics.

		N	%
Case	Valid	165	100.0
	Excluded (a)	0	0.0
	Total	165	100.0
a. This list-wise deletion is based on all variables in the procedure.			
	Cronbach’s Alpha	Cronbach’s Alpha	Items
		Based on Standardized Items	
Project	0.922	0.923	12
Equipment	0.909	0.909	5
Process	0.898	0.899	6
Organization	0.973	0.973	3

The Kaiser–Meyer–Olkin (KMO) test was adopted to assess sampling adequacy for factor analysis at a statistical range of 0–1. Calculated values higher than the minimum acceptable value of 0.5 are considered satisfactory. Table 8 shows the calculated value at 0.943, with significance. Bartlett’s test of sphericity is widely used to test for appropriateness. Thus, the KMO test and Barlett’s test of sphericity were employed to examine the suitability of data for principal component analysis [70] and cluster analysis [71].

**Table 8:** Results of the KMO and Bartlett’s tests.

		KMO and Bartlett’s Score
Kaiser–Meyer–Olkin measure of sampling adequacy		.943
Bartlett’s test of sphericity	Approx. chi-square	4306.612
	df	325
	sig.	.000

## 5. Discussion

To address RQ1, manufacturing enterprises should adopt a suitable maturity assessment model for assessing their strengths and weaknesses, enabling them to implement smart transformation projects while introducing smart manufacturing in the face of a competitive environment. Nikkhou et al. [72] explained that focusing on maturity is a guidance method for conducting an analysis of an organization’s strengths, weaknesses, and opportunities. Adopting a maturity model can guide project managers in the process of transformation. Manufacturing enterprises must develop transformation plans with support from top management at the project execution level. The vision of a company’s top management also has a positive effect on project purpose and success and is considered the vision of the project leadership team [73]. Enterprises can develop their own ideas and strategies for transitioning to smart manufacturing and identify potential crises and opportunities, effectively forecast demand, and establish control mechanisms. These measures can enable enterprises to digitally transform the benefits of management investment maximization.

Project management plays a crucial role in the implementation of new manufacturing systems and can guide the adoption of hardware and software systems [39]. Enterprises are increasingly adopting project management as a tool for enhancing productivity [74] and focusing on project benefit realization [75]. Project management plays a critical role in the transformation project plan, and thus influences the successful delivery of projects. Project managers must measure each step involved in the project and self-assess internal potential risks in project management, equipment, organization, and process aspects for successful project delivery. Project managers must evaluate the project status and assess maturity levels, verify the transformation processes, and evaluate current resources and capabilities to detect possible insufficiencies or problems in the processing of the transformation project. In the management of manufacturing transformation projects, potential problems such as design changes should be identified as early as possible at the design stage to reduce manpower reallocation and schedule control in the manufacturing stage. These factors affect the project success rate. In our survey, most enterprises agreed that project management was one of the critical processes for project managers to secure resources and budgets with the support of top management. Therefore, we incorporated project management into our project-based maturity model to enable the assessment of potential crises and opportunities for enterprises (Fig 1).

In an attempt to answer RQ2, the study performed a survey on 165 Taiwanese enterprises and used cluster analysis to categorize them into three groups to explore the key influencing factors and transformation problems related to the maturity assessment of the project, equipment, process, and organization aspects of the enterprises. The grouping was performed on the basis of industry feature and application as follows: group 1: system integration, design-oriented SMEs, and mechanical equipment; group 2: traditional manufacturer (including parts, component, accessory, plastic, and metal processing); group 3: EMS, information service providers and automation. Our analysis revealed that 59% of the enterprises considered adopting external system solutions to assist in the implementation of transformation projects and 55% reported that their current transformation project does not meet their requirements because the project process was too complex and implementation steps were not clearly illustrated. Enterprises may seek guidance from external consultants to obtain development technologies, increase visibility, and take advantage of the trend to obtain resources to further invest in industrial transformation and use the opportunity for smart transformation to retain core technologies, thereby overcoming the challenges arising out of a shortage of human resources. Moreover, the surveyed enterprises reported that technical expertise and budget are critical factors influencing the execution of transformation projects. Hence, enterprises should use external project transformation plans to meet their current project requirements and consider important influencing factors of expertise and budget in their next step toward smart transformation.

As a result, based on our data and literature analyses, our study aimed to advance research on project management and maturity models in smart manufacturing transformation to fill this gap. Our study developed a project-based maturity model providing an evaluation and assessment framework that enables organizations to identity their best practices and those of their rivals to design an optimal path to implementation. We validated the project-based maturity model as being suitable for measuring maturity levels in real environments for Taiwan-based enterprises. This model allows enterprises to identify factors influencing project management implementation and helps make priority improvements within the enterprise. For manufacturing enterprises, smart transformation is not as simple as the digitization of manufacturing process. When cost is no longer the only competitive advantage, creating a “transformation value” becomes crucial. Enterprises should use technological tools or external resources to achieve smart transformation. For this purpose, enterprises can adopt the proposed maturity model (Fig 1) to analyze their maturity levels and then identify key areas that must be strengthened to achieve a competitive edge. Therefore, it is essential to build the foundation for the operations and expertise of enterprises and then extend the service process to further innovate the products and business models.

## 6. Conclusions

In the era of digitalization, the business and manufacturing environments are on the cusp of transformation and reconstruction. The implementation of smart manufacturing is challenging for enterprises because it requires a continuous strategic commitment to attain an appropriate level of maturity and improvement of organizational capabilities in terms of project management execution, technical equipment introduction, and process optimization. This commitment yields important benefits and value creation for enterprises, especially in terms of production efficiency, increased quality, and short lead time to market. Moreover, smart manufacturing is expected to stimulate sales growth, enlarge market opportunities, and increase profitability of enterprises. The purpose of this study was to examine challenges in the smart transformation of enterprises. First, enterprises should choose a suitable maturity model as an assessment tool for self-evaluation, which can help them transform their capabilities to face the competitive environment. Enterprises must prioritize the introduction of smart transformation plans into their operational needs and then implement them step by step to achieve smart transformation goals. After the assessment, enterprises will be better equipped to handle the changing competition in the business environment and employ practical strategies involving digital technologies.

Our study highlights that project management plays a crucial role in manufacturing transformation. In addition, this study contributes to the literature in the context of smart manufacturing by proposing a project-based maturity assessment model that incorporates project management to assist enterprises in assessing their levels of transformation projects and developing strategies toward achieving smart manufacturing. The proposed assessment model comprises four blocks and 11 pillars of items identified from the extant literature and through our online survey. The proposed maturity assessment model is useful for project managers in the practical analysis of the project stages. It enables project managers to monitor the progress of the project at each stage of its implementation process and identify any requirement for resource allocation and capability improvement. This study evaluated the reliability and validity of the proposed model in enterprise transformation. Through assessment using this maturity model, enterprises can transform their processes by diagnosing potential issues and accordingly defining strategies toward achieving smart manufacturing.

Our analysis revealed that most of the surveyed enterprises were at the immature or mid-mature levels, and they planned and executed transformation projects in 2021. Few enterprises exhibited the matured level. Compared with 2018, more enterprises are actively investing in smart manufacturing transformation in 2021, and the proportion of participation has significantly increased. Thus, these findings imply a positive effect on industrial development in Taiwan.

Another crucial finding is that in the evaluation of transformation plans, enterprises preferred to use external system solutions to replace existing projects. This may be attributed to the complexity of the existing project design and the difficulty in implementation caused by unstructured explanations. Enterprises believed the main challenge to be the shortage of technical expertise, funds, and resources, which are key factors affecting the success of the transformation plan. Therefore, based on our literature and practical analyses, the project-based maturity model is suitable for Taiwan-based enterprises for evaluating their level of maturity and project plans.

However, the crisis of the COVID-19 epidemic has disrupted not only the global social order but also the business development foundation of the manufacturing industry. Many enterprises are facing the impact of the collapse of their main customers or drastic changes in the supply system. However, this epidemic can be seen as an opportunity. Enterprises can identify existing operational challenges and capacity gaps and then establish future manufacturing strategies using the proposed maturity model as a digital tool to overcome these bottlenecks and hidden barriers [76]. This will enable them to accelerate smart transformation in the project, equipment, process, and organization aspects. In such rapidly changing situations, in addition to carefully devising a situation adjustment strategy, Taiwanese manufacturers must utilize this opportunity to implement transformation plans to maintain their competitive edge in the new situation of the manufacturing industry and be better equipped in the post-pandemic era.

This study had some limitations. First, the questionnaire survey was conducted only among Taiwanese enterprises that were invited to complete, thus limiting further findings from different countries. Second, only manufacturing-based enterprises were considered in this study. Future studies can explore issues related to smart transformation in other industries. Third, this study focused only on the concept of maturity models. Future studies should also explore the relationship between other management strategies and the implementation of transformation projects to further guide enterprises in making informed decisions. Nevertheless, this study provided deeper insights into the impact of maturity assessment on transformation plans in terms of project, equipment, process, and organization and its impact on enterprises’ decision to adopt smart manufacturing projects. Furthermore, the aforementioned findings and arguments have broad implications for smart manufacturing literature and practice.

## Supporting information

S1 File (XLS)
